# Surgical Data and Early Postoperative Outcomes after Minimally Invasive Lumbar Interbody Fusion: Results of a Prospective, Multicenter, Observational Data-Monitored Study

**DOI:** 10.1371/journal.pone.0122312

**Published:** 2015-03-26

**Authors:** Paulo Pereira, David Buzek, Jörg Franke, Wolfgang Senker, Arkadiusz Kosmala, Ulrich Hubbe, Neil Manson, Wout Rosenberg, Roberto Assietti, Frederic Martens, Giovanni Barbanti Brodano, Kai-Michael Scheufler

**Affiliations:** 1 Neurosurgery, Centro Hospitalar S. Joao, Porto, Portugal; 2 Karvinska hornicka nemocnice, Karvina, Czech Republic; 3 Klinikum Dortmund, Dortmund, Germany; 4 Klinikum Amstetten, Amstetten, Austria; 5 Klinikum Kulmbach, Kulmbach Bayern, Germany; 6 Universitatsklinikum Freiburg, Freiburg, Germany; 7 Orthopaedic Surgery, Horizon Health Network, Saint John, NB, Canada; 8 Franciscus Ziekenhuis, Rosendaal, The Netherlands; 9 Fatebenefratelli Hospital, Milano, Italy; 10 Onze Lieve Vrouw Ziekenhuis, Aalst, Belgium; 11 Istituti Ortopedici Rizzoli, Bologna, Italy; 12 Hospital zum Heiligen Geist Kempen, Kempen, Germany; University of Michigan, UNITED STATES

## Abstract

Minimally invasive lumbar interbody fusion (MILIF) offers potential for reduced operative morbidity and earlier recovery compared with open procedures for patients with degenerative lumbar disorders (DLD). Firm conclusions about advantages of MILIF over open procedures cannot be made because of limited number of large studies of MILIF in a real-world setting. Clinical effectiveness of MILIF in a large, unselected real-world patient population was assessed in this Prospective, monitored, international, multicenter, observational study. Objective: To observe and document short-term recovery after minimally invasive interbody fusion for DLD. Materials and Methods: In a predefined 4-week analysis from this study, experienced surgeons (≥30 MILIF surgeries pre-study) treated patients with DLD by one- or two-level MILIF. The primary study objective was to document patients’ short-term post-interventional recovery (primary objective) including back/leg pain (visual analog scale [VAS]), disability (Oswestry Disability Index [ODI]), health status (EQ-5D) and Patient satisfaction. Results: At 4 weeks, 249 of 252 patients were remaining in the study; the majority received one-level MILIF (83%) and TLIF was the preferred approach (94.8%). For one-level (and two-level) procedures, surgery duration was 128 (182) min, fluoroscopy time 115 (154) sec, and blood-loss 164 (233) mL. Time to first ambulation was 1.3 days and time to study-defined surgery recovery was 3.2 days. Patients reported significantly (*P* < 0.0001) reduced back pain (VAS: 2.9 vs 6.2), leg pain (VAS: 2.5 vs 5.9), and disability (ODI: 34.5% vs 45.5%), and a significantly (*P* < 0.0001) improved health status (EQ-5D index: 0.61 vs 0.34; EQ VAS: 65.4 vs 52.9) 4 weeks postoperatively. One adverse event was classified as related to the minimally invasive surgical approach. No deep site infections or deaths were reported. Conclusions: For experienced surgeons, MILIF for DLD demonstrated early benefits (short time to first ambulation, early recovery, high patient satisfaction and improved patient-reported outcomes) and low major perioperative morbidity at 4 weeks postoperatively.

## Introduction

Conventional open technique for instrumented lumbar fusion requires extensive intraoperative dissection and retraction of paraspinal musculature.[1,2] Minimally invasive surgery (MIS) is intended for decreased manipulation of surrounding tissues [3] by reducing tissue damage/retraction and preserving the paraspinal and abdominal musculature during fusion procedures. From a safety perspective, MIS minimizes blood loss and reduces perioperative morbidity and infection. Ultimately, these benefits may translate into accelerated post-interventional recovery and return of function, with reduced analgesic requirements. [4,5]

Systematic reviews show potential benefits of minimally invasive instrumented lumbar interbody fusion (MILIF) over the traditional open technique.[4,5,6,7,8] However, the effectiveness and safety cannot be concluded due to the following study limitations: varied surgeon experience with MILIF [4,9] low number of patients, heterogeneity of procedures [2,6,7,10] and a non-standardized collection of surgical parameters and/or clinical outcome measures. [7,8,11]

The objective of this multicenter, observational study was to prospectively observe and document short term recovery by evaluating a standard set of short- and long-term data from a large number of patients undergoing minimally invasive posterior lumbar interbody fusion (MPLIF) or minimally invasive transforaminal lumbar interbody fusion (MTLIF) to confirm the safety and effectiveness of these techniques when performed by experienced users. We present the 4-week results from the MASTERS-D study, assessing the minimally invasive PLIF or TLIF technique for the treatment of the degenerative lumbar spine in a broad and heterogeneous multinational patient population.

## Material and Methods

### Study design and patients

MASTERS-D was a large prospective, monitored, international 1-year observational study (Study registered at ClinicalTrial.gov NCT01143324) in patients with degenerative lumbar spine causing back and/or leg pain.

It was conducted in 19 centers across 14 countries (EU, Canada, and Middle East). All participating surgeons were required to have performed ≥30 pre-study MILIF procedures for degenerative lumbar spine indications. All patients received routine standard of care according to hospital protocol. Study sites, which had the Oswestry Disability Index (ODI) [12, 13] and visual analog scale (VAS) [14] back and leg pain questionnaires incorporated into their routine practice, were selected. This observational study did not add to the patients’ burden of illness or risks. All patients signed an informed consent/patient-release form before study inclusion. Data collection was anonymized.

Time from the start of patient recruitment until last patient last visit was from June 24^th^ 2010 until October 2^nd^ 2012.

### Ethics approval

This study was carried out in compliance with the Declaration of Helsinki laws and regulations of the countries in which the studies were conducted. Ethics Committee (EC)/Institutional Review Board (IRB)/Human Research Ethics Committee (HREC)/Data protection authority/Competent Authority approvals received are described in Table 1.

**Table 1:** Ethical approvals.

Hospital	City/Country	Ethics Committee (EC)/Institutional Review Board (IRB)/Human Research Ethics Committee (HREC)/Data Protection Authority
**Klinikum Amstetten**	Amstetten, Austria	Ethikkommission für das Bundesland Neiderösterreich am Sitz des Amtes der NÖ Landesregierung, abteilung Sanitäts-undKrankenanstaltenrecht, Landhausplatz 1, Haus 15b, 3109 St. Pölten, Austria
**O.L.V. Aalst**	Aalst,Belgium	Local Ethisch Comité, O.L. Vrouwziekenhuis Aalst, Moorselbaan 167, 9300 Aalst, Belgium. Submission to the DataProtection Authority
**Horizon Health Network; East Spine Centre**	Saint John, Canada	Local Research Ethics Board,Horizon Health Network, 5DN SJRH, 400 University Avenue, Saint John, New Brunswick, Canada E2L 4L2
**Karvinska hornicka nemocnice, a.s**	Karvina, Czech Republic	Ethics Committee approval not required by local regulatory agencies, investigator statement collected and confirmed by the investigator
**Klinikum Kulmbach**	Kulmbach,Germany	Ethics Committee approval not required by local regulatory agencies, investigator statement collected and confirmed by the investigator
**Universitätsklinikum Magdeburg**	Magdeburg, Germany	Ethics Committee approval not required by local regulatory agencies, investigator statement collected and confirmed by the investigator
**Universitätsklinikum Freiburg**	Freiburg,Germany	Central ethics committee (Albert–Ludwigs- Universität Freiburg—Ethikkommission, Engelbergerstrasse 21, 79106 Freiburg, Germany
**Marienhaus-Klinikum**	Bendorf,Germany	Ethics Committee approval not required by local regulatory agencies, investigator statement collected d and confirmed by the investigator
**Mediterraneo Hospital**	Glyfada, Greece	Local Ethics Committee,Mediterraneo Hospital, Athens, Greece. Notification to Competent Authority; Submission to the Data Protection Authority
**The Tel Aviv Sourasky Medical Center**	Tel Aviv,Israel	Local Ethics Committee TelAviv Sourasky Medical Center, Tel Aviv, Israel and Hospital director
**Istituti Ortopedici Rizzoli**	Bologna,Italy	Istituto Ortopedico Rizzoli diBologna, Via Di Barbiano, 1/10-40136 Bologna, Italy
**Fatebenefratelli Hospital**	Milano,Italy	Comitato Etico Scientifico,Ospedale Fatebrenefratelli e oftalmico, Azienda ospedaliera di rlievo nazionale, Coso di Pora Nuova, 23–20121 Milano, Italy
**Franciscus Ziekenhuis Roosendaal**	Roosendaal,The Netherlands	Ethics Committee approval not required by local regulatory agencies, investigator statement collected d and confirmed by the investigator
**Bergman Clinics**	Naarden, The Netherlands	Ethics Committee approval not required by local regulatory agencies, investigator statement collected d and confirmed by the investigator
**Medical University of Gdańsk**	Gdansk, Poland	Local Independent Medical EthicCommittee for Scientific Researches at Gdansk Medical University, M. Sklodowskeij-Curie 3a, St., 80–210 Gdansk, Poland
**Hospital S. João**	Porto,Portugal	Local Comissão de Ética doHospital S. João, EPE Alameda Prof. Hernani Monteiro, 4200–319 Porto, Portugal. Submission to the Data Protection Authority
**Ustredna vojenska nemocnica SNP**	Ruzomberok, Slovakia	Etická Komisia,Ustrednex Vojenskej Nomocnice SNP Ruzomberok, Gen Milosa Vesela 21, 034 26 Ruzomberok,Slovakia
**Hospital Clínic de Barcelona**	Barcelona, Spain	Comité de Investigación delHospital Clínic de Barcelona, Hospital Clínic de Barcelona, Vallarroel, 170–08036 Barcelona, Spain
**Guys & St. Thomas NHS Trust**	London,United Kingdom	Local NHS National Research Ethics Service, South West London REC 3, Room4W/12 4 Floor West, Charing Cross Hospital, Fulham Palace Road, London W6 8RF, UK
**London Bridge Hospital**	London, United Kingdom	Ethics Committee approval not required by local regulatory agencies, investigator statement collected d and confirmed by the investigator

### Eligibility criteria

To attain a patient population that is reflective of the “real world,” eligibility criteria were broad and based on the approved indications for the devices used in this study. Eligible patients over 18 years of age were clinically assessed as requiring a single- or double-level instrumented lumbar fusion for the treatment of the degenerative lumbar spine. Patients were planned to undergo the fusion procedure using MPLIF or MTLIF techniques and to receive a CD HorizonSpinal System (Medtronic Sofamor Danek USA, Inc.) via the MAST approach (Minimal Access Spinal Technologies, Medtronic Sofamor Danek USA, Inc.), in accordance with the device-labeling. Patients who had previously undergone open lumbar spine surgery other than microdiscectomy were excluded from the study, as were patients with indications for the procedure other than degenerative spine disease.

### Surgical procedures

Definitions according to study protocol are presented here. In the open lumbar procedure, a midline approach requiring partial or complete detachment of the lumbar fascia and paraspinal muscles was used to address the spinal pathology and placement of instrumentation. The minimally invasive procedure was defined as a muscle sparing surgical technique using an intermuscle or transmuscle splitting approach that minimizes detachment of the lumbar fascia and paraspinal muscles to address the spinal pathology and placement of instrumentation. In the mini-open technique, instruments were placed using direct vision of target structures via an intermuscle or transmuscle splitting approach. In the percutaneous technique, instrumentation was placed using radiographic or navigation guidance via stab incisions without direct vision of target structures.

The muscle sparing minimally invasive approach could be performed unilaterally or bilaterally for instrumentation and spinal decompression, at the investigator’s discretion. One or two cages were placed in the intervertebral space to maintain or restore disc height. To achieve interbody fusion, bone grafts and/or substitutes were used. The posterior stabilization of the treated spinal segments was performed using the CD Horizon Spinal System.

### Protocol deviations

From baseline at which time enrolment took place, until the 4-week follow up time point, 37 protocol deviations were reported, 33 of which occurred at the baseline visit. The protocol deviations listed were: wrong version of the Informed Consent Form (ICF) initially used, patient informed consent process not entirely clear, patients enrolled prior to the date the Clinical Trial Agreement (CTA) was fully executed, calculated BMI above 40, two Serious Adverse Events (SAE) were found not to have been reported within 24 hours, site began enrolling after site initiation but before actual site activation. Where possible (e.g. correct ICF version to be used) the deviations were corrected and site training was provided as corrective measure to further prevent these protocol deviations.

### Study assessments

Data were collected prospectively as per standard of care and included medical history, preoperative data, assessment of time from surgery to first ambulation, time to the surgery recovery day (SRD), and time to discharge. The objective of the SRD assessment is to evaluate when the patient could be discharged based on his actual clinical condition because the effective day of discharge may be prolonged by factors other than the patient's clinical recovery such as social factors, reimbursement schemes, and/or hospital protocol. Postoperative follow-up visits were performed according to standard hospital routine, with the recommended schedule including a visit at 4 weeks and 3, 6, and 12 months. (Table 2) Patient-reported outcomes (PRO) (ODI [12, 13], back and leg pain VAS intensity scores [14], EQ-5D [15] and patient satisfaction were collected. Back and leg pain were assessed on a 10-cm VAS scale (where 0 = no pain, 10 = the worst possible pain) preoperatively, during the hospital stay at day 2 after surgery, at surgery recovery day, at discharge, and at 4 weeks postoperatively. Disability was rated on a 0% to 100% scale preoperatively and postoperatively at 4 weeks using the 10-item ODI (where 0% = minimal disability and 100% = maximal disability). Health-related quality-of-life was assessed using the five-item EQ-5D questionnaire, if part of the standard care at the study center, with three levels for each dimension: no problems, some problems, or extreme problems. EQ-5D index scores were obtained using the UK population value set (http://www.euroqol.org). Patients then also completed the EQ VAS to self-rate their overall health state on a 0 to 100 scale (where 0 = maximal health-related problems and 100 = minimal health-related problems). Patient satisfaction as assessed by the surgeon.

**Table 2:** Summary of MASTERS-D scheduled procedures and assessments.

Procedures/Assessments		Pre-op	Day ofSurgery (D0) (1)	Hospital stay and discharge (2)			Follow-up visits after the surgery			
				D2	SRD	DIS	4(±2)week	3(±1) month	6(±1)month	12(±2) month
**Data release form OR informed consent form**		**X**								
**Demographics**		**X**								
**Medical history**		**X**								
**Surgery indication**		**X**								
**Pain medication**		**X**		**X**	**X**	**X**	**X**	**X**	**X**	**X**
**MRI (or CT Scan)**		**X**								**X (3)**
**PatientQuestionnaires**	**VAS (4)**	**X**		**X**	**X**	**X**	**X**	**X**	**X**	**X**
	**ODI**	**X**					**X**	**X**	**X**	**X**
	**EQ-5D (3)**	**X**					**X**	**X**	**X**	**X**
**Rehabilitation program**							**X**	**X**	**X**	**X**
**Work status**		**X**					**X**	**X**	**X**	**X**
**X-rays (AP, lateral, F/E)**		**X**								**X (3)**
**Surgery and hospital data**			**X**	**X**	**X**	**X**				
**Patient’s satisfaction**							**X**	**X**	**X**	**X**
**Adverse events**			**X**	**X**	**X**	**X**	**X**	**X**	**X**	**X**

Postoperative follow-up visits were performed according to standard hospital routine, with the recommended schedule including a visit at 4 weeks and 3, 6, and 12 months. Assessment of time from surgery to first ambulation (defined as the number of days after surgery before patients were able to get out of bed and ambulate with or without assistance), time to the Surgery Recovery Day (defined as the number of days after surgery until patients no longer needed intravenous infusion of analgesic drugs, had no surgery-related complications/AEs impeding discharge, and no longer needed nursing care) and time to discharge. (1) Day ‘0’ (D0) is the day of the surgery, D1 is the first day after surgery, D2 the second and so on. (2) SRD = Surgery Recovery Day; DIS = Day of Discharge, (3) Optional, only if it is routine practice (standard of care) in the center. (4) VAS scores for back and leg pain intensity.

### Endpoints and statistical analysis outputs

The primary objective was to observe and document the patients’ short-term recovery after surgery. The primary endpoints were a) the time to first ambulation after surgery and b) the time to SRD.

The intent was to perform descriptive and exploratory data analyses, with calculations of summary statistics to report estimations and confidence intervals of endpoints of interest. Approximately 200 patients were planned to be enrolled with a minimum of 150 and maximally up to 250 patients. No sample size calculations were performed.

Secondary endpoints included changes from baseline in PROs over time, as well as documentation of adverse events (AEs). Interim analysis of short-term data up to 4 weeks (±2 weeks) postoperatively was prespecified in the study protocol. In the present analysis, 4-week data are based on data collected between discharge and 8 weeks postoperatively (in order to account for standard practices differing slightly from the recommended schedule in some centers).

Adverse Event (AE) and Serious Adverse Event (SAE) were defined, respectively, as “Any untoward medical occurrence in a subject”, and “An AE that led to death; led to serious deterioration in the health of a subject; led to fetal distress, death or congenital abnormality”. All investigators classified the AEs in seriousness and relatedness to the surgery, MAST approach, device (unrelated, unlikely, possibly, probably or definitely). For reporting purposes, all adverse events were classified into Lowest Level Terms following Medical Dictionary for Regulatory Activities Terminology (MedDRA) version 15.1.

For continuous variables, summary statistics were calculated, together with the number of missing and non-missing values. For categorical variables, absolute and relative frequencies (based on non-missing values) were provided. The change in each PRO score was calculated for all patients with a valid observation of both the preoperative and 4-week score of the PRO in concern. Changes from baseline in PROs at 4 weeks postoperatively were analyzed (depending on the Shapiro-Wilk test results on normality) using the two-sided *t* test for paired comparisons and the Wilcoxon signed rank test with a significance level of 0.05. Results are presented as means (± standard deviation).

Analyses were carried out including all available data from the patients submitted to a MAST procedure. In case of missing values for a certain variable, N was equally reduced. Data from three patients lost to follow up were included up to the time that data were no longer obtainable. Baseline data and surgery data were included, however change from baseline for the PRO’s could not be obtained from the three patients lost to follow up.

Clinical success rates for VAS back pain and ODI were calculated according to Ostelo *et al*., [16] who proposed minimal important change (MIC) values of 15 for the VAS (0–100), and 10 for the ODI (0–100).

Surgeries performed in this study were carried out by surgeons experienced in the procedure. While this minimizes the possibility that primary endpoints could be prolonged due to inexperience of the surgeon, at the same time it means that data are not 100% applicable to the real situation where surgeons that not necessarily have experience with the procedure can also perform it.

## Results

### Patient disposition and baseline demographics

**Fig 1:**
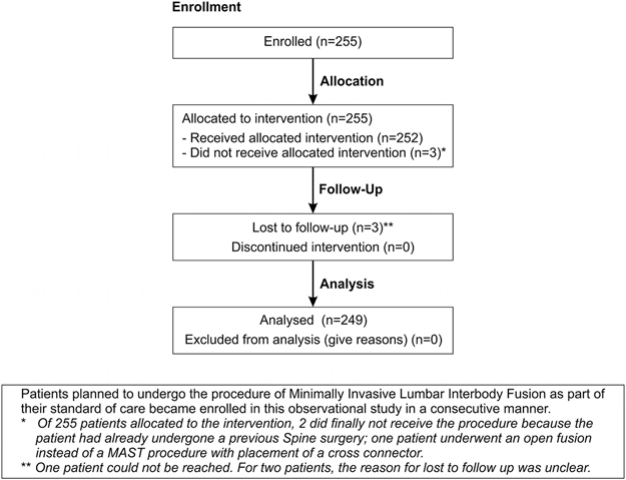
Flow diagram of patient follow up.

A total of 255 patients were enrolled in the study but three did not undergo the procedure due to different reasons (Fig. 1). A total of 252 patients underwent a MILIF procedure of which 249 (99%) patients remained in the study at 4 weeks postoperatively. From the total population, 56.3% were female; mean age was 53.8 ± 11.8 years; body mass index was 27.7 ± 4.6 (kg/m^2^); duration of symptoms resulting in planned surgery was 28.5 ± 38.2 months, and main indications for surgery were leg pain (52.0%), back pain (38.9%), and neurogenic claudication (9.1%). Patient demographics of one- and two-level subgroups are shown in detail in Table 3. Preoperative degenerative lumbar pathologies shown in Table 4, disc pathology (93.7%), stenosis (71.4%) and spondylolisthesis (52.8%) were the commonest pathologies.

**Table 3:** Patient Demographics.

Characteristic	Total population	One-level operated subgroup	Two-level operated subgroup
**Total number of patients**	252	210 (83.3%)	42 (16.7%)
**Gender, n (%):**			
** Female**	142 (56.3)	123 (58.6)	19 (45.2)
** Male**	110 (43.7)	87 (41.4)	23 (54.8)
**Age, mean ± SD (y)**	53.8 ± 11.8	52.8 ± 11.9	58.7 ± 9.9
**BMI, mean ± SD (kg/m^2^)**	27.7 ± 4.6	27.5 ± 4.5	28.4 ± 5.2
**Duration of symptoms resulting in planned surgery, mean ± SD (months)**	28.5 ± 38.2	27.7 ± 39.5	32.5 ± 31.3
**Duration of conservative treatment, mean ± SD (months)**	20.7 ± 34.3	20.9 ± 35.6	19.5 ± 27.4
**Pre-existing conditions relevant to study, n (%)**	94 (37.3)	74 (35.2)	20 (47.6)
**Patients with previous lumbar surgeries, n (%)**	47 (18.7)	39 (18.6)	8 (19.0)
**At target level, n (%):**			
** Microdiscectomy (Open)**	8 (3.2)	5 (2.4)	3 (7.1)
** Microdiscectomy (MIS)**	23 (9.1)	20 (9.5)	3 (7.1)
** Decompression (MIS)**	9 (3.6)	7 (3.3)	2 (4.8)
**Main indication for surgery, n (%):**			
** Back pain**	98 (38.9)	85 (40.5)	13 (31.0)
** Leg pain**	131 (52.0)	110 (52.4)	21 (50.0)
** Neurogenic claudication**	23 (9.1)	15 (7.1)	8 (19.0)

*MIS*, *minimally invasive surgery*.

**Table 4:** Preoperative Degenerative Lumbar Pathologies.

Pathology, n (%)	Number (N = 252)
Disc pathology:	236 (93.7)
Disc reduction <50%	124 (49.2)
Disc reduction ≥50%	94 (37.3)
Disc protrusion	109 (43.3)
Disc extrusion	28 (11.1)
Disc sequestration	11 (4.4)
Stenosis:	180 (71.4)
Central/lateral recess	116 (46.0)
Foraminal	74 (29.4)
Not specified	5 (2.0)
Spondylolisthesis:	133 (52.8)
Degenerative	92 (36.5)
Isthmic	42 (16.7)
Grade 1	112 (44.4)
Grade 2	21 (8.3)
Grade >2	0 (0)
Other	21 (8.3)

### Surgical procedures performed

From the 252 patients, 12 were submitted to a PLIF surgery and 239 to a TLIF approach. One patient underwent at 1^st^ level a PLIF and at the 2^nd^ level a TLIF. Eighty three per cent received one-level surgery and 16.7% received two-level surgery, details on the operated levels are presented in Table 5. As expected, the duration of two-level surgery (182 min) was longer than one-level surgery (128 min) and mean operative blood loss per patient was greater (one level: 163.9 ± 139.7 mL vs two levels: 233.1 ± 229.0 mL) (Table 5). Only one patient (0.4%) required blood transfusion in the two-level group. Surgical approaches varied substantially as shown in Table 6.

**Table 5:** Intraoperative Surgery Results.

	Total population (n = 252)	One-level operated subgroup(n = 210)	Two-level operated subgroup (n = 42)
Surgery duration, mean ± SD (min)	136.8 ± 50.4	127.7 ± 43.5	182.0 ± 58.3
Fluoroscopy duration, mean ± SD (sec)	122.0 ± 130.7	115.1 ± 123.9	154.1 ± 156.6
Blood loss, mean ± SD (mL)	175.8 ± 160.1	163.9 ± 139.7	233.1 ± 229.0
Blood transfusion needed, n (%)	1 (0.4)	0	1 (2.4)
One level, %	210 (83.3)	210 (83.3)	
L2–L3	5 (2.0)	5 (2.0)	
L3–L4	15 (6.0)	15 (6.0)	
L4–L5	104 (41.3)	104 (41.3)	
L5–S1	86 (34.1)	86 (34.1)	
Two levels, %	42 (16.7)		42 (16.7)
L3–L4 and L4–L5	11 (4.4)		11 (4.4)
L4–L5 and L5–S1	31 (12.3)		31 (12.3)

**Table 6:** Mini-Open Surgical Approaches and Decompression and Fixation details.

Surgical approach (first level), n (%)	Number of patients n = 252 (%)
Unilateral approach:	156 (61.9)
No decompression	46 (18.3)
Unilateral decompression	92 (36.5)
Bilateral decompression (over the top)	18 (7.1)
Bilateral approach:	96 (38.1)
No decompression	13 (5.2)
Unilateral decompression	62 (24.6)
Bilateral decompression:	21 (8.3)
Over the top	5 (2)
From both sides	16 (6.3)
Posterior Fixation Techniques, n (%):	
Bilateral, percutaneous	123 (48.8)
Bilateral, mini-open	100 (39.7)
One side mini-open + one side percutaneous	24 (9.5)
Unilateral percutaneous	4 (1.6)
Unilateral mini-open	1 (0.4)

### Primary endpoints

The mean time to first ambulation was 1.3 ± 0.5 days across the 252 patients and was similar among patients who underwent one- (1.3 ± 0.5 days) or two- (1.4 ± 0.5 days) level surgery. Similarly, the mean time to the SRD was 3.2 ± 2.0 days in the 252 patients, with recovery times being similar between patients operated on one (3.2 ± 2.1 days) and two (3.1 ± 1.5 days) levels. The mean time to discharge was 6.3 days across all 252 patients, 6.1 or 7.4 days in patients operated on one or two levels, respectively.

### Patient-reported outcomes

**Fig 2:**
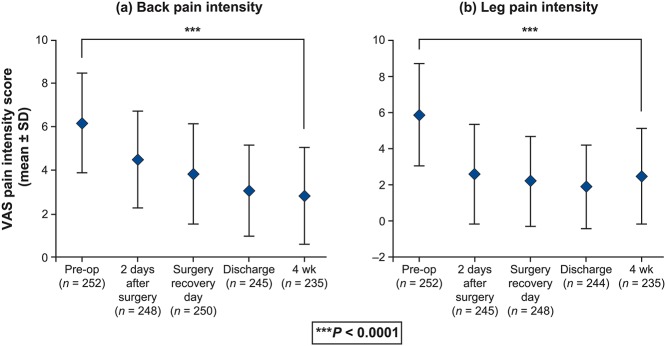
Back pain intensity (a) and leg pain intensity scores (b) reported preoperatively and postoperatively on a 10-cm visual analog scale (VAS), where 0 = minimal pain intensity or pain frequency and 10 = maximal pain intensity or pain frequency (total population; N = 252). *P* < 0.0001 for difference between preoperative (B: 6.2 ± 2.3; L: 5.9 ± 2.8), and 4-week postoperative scores (B: 2.9 ± 2.2; L: 2.5 ± 2.6).

**Fig 3:**
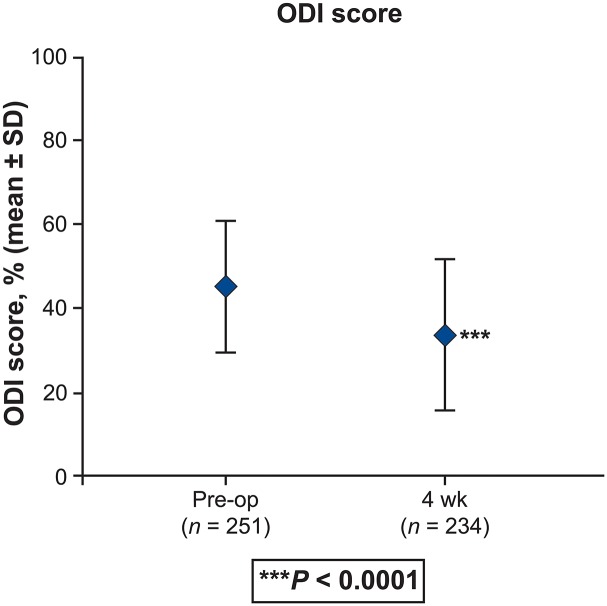
Oswestry Disability Index (ODI) scores reported preoperatively and 4-week postoperatively on a 0% to 100% scale, where 0% = minimal disability and 100% = maximal disability (total population; N = 252). *P* < 0.0001 for difference between preoperative (45.5 ± 15.4) and 4-week postoperative scores (34.5 ± 17.3).

**Fig 4:**
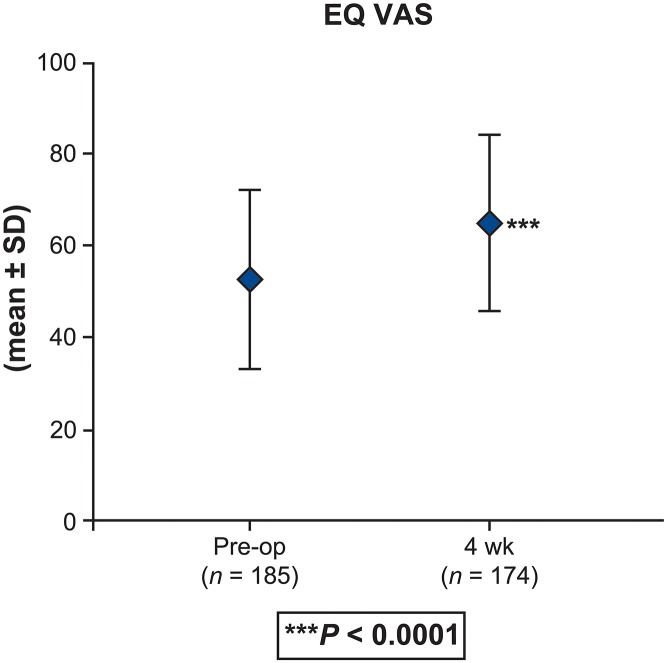
EQ VAS scores reported preoperatively and 4-week postoperatively on a 0 to 100 scale, where 0 = maximal health-related problems and 100 = minimal health-related problems (total population; N = 252). *P* < 0.0001 for difference between preoperative (52.9 ± 19.5) and 4-week postoperative scores (65.4 ± 18.6).

**Fig 5:**
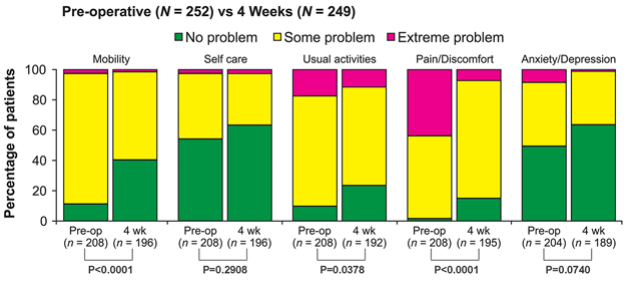
Five individual EQ-5D domains were reported preoperatively and 4-week postoperatively. At 4 weeks, the percentages of patients who reported no health-related problems increased from preoperative values in each domain: mobility (40.3% vs 11.1%), self-care (63.3% vs 54.3%), usual activities (23.4% vs 9.6%), pain/discomfort (14.9% vs 1.4%), and anxiety/depression (63.5% vs 49.5%).

Statistically significant (*P* < 0.0001) and clinically meaningful changes from baseline in PROs were reported postoperatively at 4 weeks. [20] There was an improvement in back and leg pain intensity (by 3.2 and 3.4 points, respectively) (*P* < 0.0001) (Fig. 2). Improvement of disability is demonstrated by a 10.6-point reduction in the ODI at 4 weeks (Fig. 3). Changes were similar regardless of whether patients received one- or two-level interbody fusion. The clinical success rate at 4 weeks was 51.1% with regard to ODI and 74.5% based on the back pain intensity. Improvements in EQ VAS scores of 12.0 points were noted at 4-week follow-up (*P* < 0.0001) (Fig. 4). Correspondingly, the percentage of patients reporting no health-related problems increased from preoperative values in each of the five individual EQ-5D domains: mobility (40.3% vs 11.1%, P < 0.0001), self-care (63.3% vs 54.3%, P = 0.2908), usual activities (23.4% vs 9.6%, P = 0.0378), pain/discomfort (14.9% vs 1.4%, P < 0.0001), and anxiety/depression (63.5% vs 49.5%, P = 0.0740) (Fig. 5). The EQ-5D index improved from a mean of 0.34 preoperatively to 0.61 at 4 weeks (*P* < 0.0001). Assessed by the surgeon at 4 weeks, the patient satisfaction with results of treatment post-surgery was 84.2% (192/228), 82.5% (188/228) indicated to be helped by the treatment as expected and 80.2% (183/228) would have the same treatment again for the same condition. Patients reported that 85.5% were: completely (11.8%, 27/228), much (53.5% (122/228) or slightly improved (20.2%, 46/228) with regards to their back pain recovery compared to preoperative.

### Adverse events

Within the 4-week postoperative time window, 39 AEs in 32 patients were considered by the investigator to be related to surgery or MAST or device, one of which was considered to be primarily MAST related (Table 7). Six AEs in five patients were classified as serious AEs (SAEs); all of these resolved. A limited subcutaneous abscess was experienced by one patient. Thus, the overall incidence of surgical site infection was 0.4%. No deep surgical site infections were reported. One surgical revision due to an intraspinal hemorrhage was reported. No SAEs were reported in patients who had MPLIF surgery.

**Table 7:** Adverse Events (AEs) and serious adverse events (SAEs) in Total Population.

MedDRA code low level terms	Count of MAST procedure related	Count of total related to device OR surgery OR MAST (serious event)
Acute allergic reaction		1 (1)
Back pain		4 (1)
Confusion postoperative		1 (1)
Dural tear		4
Fever		2
Hypoesthesia		3
Implant site seroma		2
Incision site abscess		1
Leg pain	1	6 (1)
Lumbar radiculopathy		3
Nausea		4
Sacro-iliac pain		1
Spinal hematoma		1 (1)
Urinary tract infection		3
Urosepsis		1 (1)
Vertigo		1
Vomiting		1
**Total**	**1**	**39 (6)**

## Discussion

This is the largest observational, prospective, multicenter study of MILIF conducted to date in patients with lumbar degenerative disorders aimed to observe and document short-term recovery. Our results indicate that minimally invasive techniques offer the potential of favorable perioperative morbidity, early clinical recovery, improvement in PROs, high patient satisfaction and low complication rates, when conducted by experienced physicians.

Based on the current literature, MILIF appeared to be at least as effective, if not superior to conventional open surgery regarding short-term clinical recovery. The time span to full ambulation of 1.3 days after MILIF compares favorably with the interval of 1 to 13.4 days reported for conventional open-surgery procedures.[1,6,10] In the current study, the mean time to protocol-defined SRD was 3.2 days and time to hospital discharge was 6.4 days. The effective day of discharge may be delayed by factors other than the patient's clinical recovery such as social factors, reimbursement schemes, and/or hospital protocol. However, these results still compare favorably to data from pertaining literature on conventional open surgery (mean times to discharge: 4.2–14.6 days). [4,6,17,18]

This is the first study to demonstrate clinically meaningful improvements in disability and back pain scores as soon as 4 weeks postoperatively in more than 50% (ODI) and 70% (back pain) of the patients, respectively. These improvements are reflected in the high levels of patient satisfaction with surgery (84.2%). Other studies have reported similar outcomes in pain VAS, ODI, and EQ-5D comparing MIS fusions with conventional open surgery. [4,17] However, the consistent and rapid amelioration in our large patient population within the first few weeks after surgery corroborate scientific evidence in favor of MIS.

In the current study, the duration of surgery conducted by the experienced surgeons was 128 and 182 minutes for one- and two-level MILIF, respectively. Although these times are comparable with those reported in the literature for open surgical procedures, they generally fall within the lower ranges cited for open surgery [1,4,7 17,18]. Perioperative safety data indicate that blood loss was low (mean: 176 mL) following MILIF when compared with open surgery. [1,7,8,17,18] In contrast, the duration of fluoroscopy (mean: 122 sec), and hence radiation exposure, were longer than the values reported for open surgery. [6,18] Performing these and other fluoroscopically guided procedures requires careful monitoring to ensure compliance with annual dose limit restrictions. [18]

The complication rate following MILIF was low in the current study, with surgical site infection rate of 0.4% at 4 weeks. This rate is comparable with other studies: 0.15% [19] 663 patients), 0.22% [20] (1213 patients), 0.9–2.6% and 1.2–7.2 [21] (37,137 patients). In contrast, higher infection rates were observed in a large study (N = 5170) reporting on MILIF vs open surgery in two-level fusion (4.6% vs 7.0%, *P* = 0.037) and one-level fusion (4.5% vs 4.8%; *P* = 0.77). [23]

Evidence suggests that MILIF surgery is similar in duration to open surgery with significantly fewer complications, when completed by experienced surgeons. [7,9,11] The current study was conducted exclusively by experienced surgeons (≥30 MILIF procedures pre-study). This was planned this way by study design to limit the possibility of surgeon experience to be of important influence on the primary outcome values “Time to first ambulation” and “Time to surgery recovery day”. At the same time, one could argue that this limits the possibilities for extrapolation of the results. In this study, complication rates were low. The results presented herein show that the level of experience required in the study design is sufficient to attain clinical benefit with MILIF with few complications. Furthermore based on the current study, the learning curve associated with mastering the MILIF technique should be manageable at most spine centers with medium patient loads.

One of the additional strengths of this study, aside from the large patient population, is the applicability of the study data to the “real-world” patient population. The eligibility criteria were broad and based on the indication for one- or two-level interbody fusion for lumbar degenerative pathologies. Elderly or obese patients were not excluded from the study, as it has been shown that neither age nor obesity should be considered contraindications for MILIF.[22] Our data show the incorporation of specific MIS techniques into each surgeon's surgical plan is highly individual with great variability among surgeons. The respect for this variability strengthened the generalizability of the study findings. Further analysis is needed to determine the influence of the different surgical techniques on clinical outcomes.

Although this is not a randomized controlled trial, which could be interpreted as a limitation to this study, data from a nonselected patient population provide valuable information concerning the effectiveness and safety of MILIF in clinical practice. In addition, longer-term data are required to assess the sustainability of the outcomes observed in this 4-week analysis. Variability in the 4-week data collection point due to the study’s observational character and in homogenous standard practices among centers (slightly differing from the recommended schedule) meant that data up to 8 weeks were included in the analysis. However, a significant impact of this adjustment on outcomes analysis is unlikely with the 95% CI of the mean duration to the visit (33.0‒35.1 days) falling into the originally recommended 4-week (±2 weeks) timeframe. The potential for long-term benefit after MILIF is evidenced by recent reports.[23] Evaluation of the full 1-year follow-up data will elucidate whether the benefits of MILIF can be maintained over mid-term in patients with degenerative lumbar disorders.

## Conclusions

For experienced surgeons, minimally invasive lumbar interbody fusion for degenerative lumbar disorders demonstrated early benefits (short time to first ambulation, early recovery, high patient satisfaction and improved patient-reported outcomes) and low major perioperative morbidity at 4 weeks postoperatively.

Support: This study was sponsored by Medtronic Spinal and Biologics. The authors confirm that all ongoing and related trials for this drug/intervention are registered. All sites received research funding from Medtronic. None of the authors received payment for writing this manuscript.
